# Cancer du sein sur tissu mammaire ectopique: à propos de 2 cas

**Published:** 2012-11-14

**Authors:** Houssam Haddad, Zouhour Bourhaleb, Tijani El Harroudi, Loubna Mezouar, Mohamed El Hfid

**Affiliations:** 1 Département de Radiothérapie, Centre régional d'oncologie, Tanger, Maroc; 2 Département de Radiothérapie, Centre régional d'oncologie, Oujda, Maroc; 3 Département de chirurgie, Faculté de médecine, Oujda, Maroc

**Keywords:** Cancer, sein, tissu mammaire ectopique, Cancer, breast, ectopic breast tissue

## Abstract

Le cancer du sein sur tissu mammaire ectopique est une tumeur rare qui représente 0,2 à 0,6% de l′ensemble des cancers du sein. Les auteurs en rapportent 2 cas pris en charge dans 2 centres d′oncologie marocains. Il s′agit de 2 patientes âgées de 31 ans et 47 ans présentant un cancer du sein sur tissu mammaire ectopique en situation axillaire. Une tumorectomie avec curage ganglionnaire axillaire homolatéral a été réalisée chez les 2 patientes dont une après une chimiothérapie néoadjuvante. Le traitement adjuvant a compris une chimiothérapie, radiothérapie puis une hormonothérapie. Après un recul de 12 mois et 20 mois respectivement, les 2 patientes sont en rémission complète.

## Introduction

La glande mammaire dérive de la crête mammaire ou ligne lactée primitive [1, 2]; une anomalie du développement embryologique peut rarement conduire à l'apparition de tissu mammaire ectopique, celui-ci peut siéger tout au long du trajet de la ligne lactée primitive principalement au niveau de la région axillaire [3]. La dégénérescence cancéreuse de ce tissu mammaire ectopique peut poser un double problème diagnostique et thérapeutique. Nous en rapportons 2 cas diagnostiqués simultanément dans 2 centres d'oncologie marocains, et à travers lesquels, nous citerons quelques particularités diagnostiques et thérapeutiques.

## Patient et observation

### Observation 1

Patiente âgée de 31 ans, célibataire, ayant une tante maternelle suivie pour un cancer du sein, qui a présenté 3 ans avant sa première consultation, une masse axillaire droite augmentant progressivement de volume. L'examen clinique a retrouvé une masse axillaire droite de 2,5 cm bilobée, indolore, mobile par rapport aux plans superficiel et profond. L'examen bilatéral des seins n'a pas permis de palper de nodule. L’échographie axillaire a montré une lésion hypoéchogène hétérogène avec atténuation postérieure suspecte de malignité. Devant ce contexte, une mammographie bilatérale s'est avérée normale. Elle a été complétée par une imagerie par résonnance magnétique (IRM) afin "d'innocenter" les seins, celle-ci était normale. Une biopsie exérèse de la masse axillaire avec curage ganglionnaire axillaire homolatéral a été réalisée. L’étude anatomo-pathologique a montré la présence de 2 nodules durs de 1,5 cm et de 0,5 cm. A la microscopie, il s'agissait d'un adénocarcinome peu différencié bordé de tissu mammaire ectopique exprimant les récepteurs hormonaux estrogéniques et progestéroniques et n'exprimant pas l'Her-2-neu, les limites d'exérèse passaient en zone saine et les 10 ganglions réséqués lors du curage ganglionnaire étaient tous envahis, Il s'agissait donc d'un adénocarcinome développé sur tissu mammaire ectopique. Un bilan d'extension comprenant une radiographie thoracique, une échographie abdomino-pelvienne et une scintigraphie osseuse n'a pas révélé de localisations secondaires pulmonaires ni hépatiques ni osseuses; la tumeur a été ainsi classé pT1c N3 M0 selon la classification TNM 2002. Le traitement adjuvant a consisté en une chimiothérapie adjuvante (3FEC100 + 3Docetaxel), puis d'une radiothérapie externe sur le sein droit, la région axillo-sus claviculaire et la chaîne mammaire interne (devant l'envahissement massif des ganglions axillaires). La patiente est actuellement sous hormonothérapie adjuvante (Tamoxifène) et maintient une rémission complète après 12 mois de la fin du traitement.

### Observation 2

**Figure 1:**
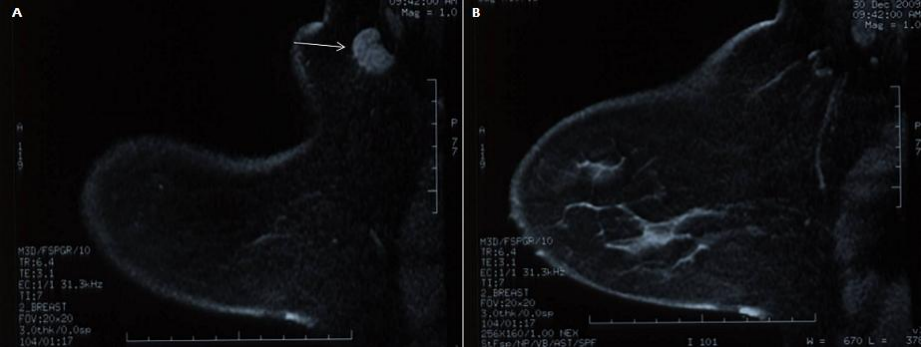
IRM Mammaire: a) Adénopathie axillaire droite (Flèche), b) le reste de la glande mammaire est indemne de toute lésion

Patiente de 47 ans, sans antécédents pathologiques particuliers, qui a consulté pour un nodule axillaire droit apparu depuis 20 mois et augmentant progressivement de volume. L'examen clinique a objectivé une adénopathie axillaire droite de 3 cm fixée à la peau. L'examen des seins était sans particularités. La mammographie bilatérale n'a pas montré de lésion mammaire, seule une adénopathie axillaire droite a été objectivée sur l'incidence oblique. Le bilan radiologique a été complété par une IRM mammaire (Figure 1) qui a montré une adénopathie axillaire droite sans retrouver de lésion mammaire. Devant ce contexte, une biopsie axillaire a conclu en une métastase ganglionnaire axillaire d'un carcinome indifférencié exprimant les récepteurs hormonaux d'origine probablement mammaire. Une chimiothérapie néoadjuvante a été alors décidée afin de permettre la résécabilité de la lésion axillaire; ainsi 3 cures du protocole AC60 suivies de 3 cures de Docetaxel ont été reçues ce qui a permis de mobiliser la masse axillaire. Ensuite, une résection de la masse axillaire droite avec curage ganglionnaire axillaire ont été réalisés. L’étude anatomo-pathologique a montré, à la macroscopie, une tumeur de 2 x 1,5 x 1 cm associée à 18 ganglions dont le plus gros mesurait 4 cm. A la microscopie, il s'agissait d'un carcinome lobulaire infiltrant de grade I développé sur une glande mammaire ectopique axillaire infiltrant le derme et l’épiderme avec images d'emblols vasculaires. Deux ganglions sur les 18 prélevés étaient envahis dont un avec rupture capsulaire. Les récepteurs hormonaux étaient exprimés sans surexpression de l'Her-2-neu. Une radiothérapie externe sur la région axillo-sus claviculaire a été délivrée avec un complément sur le lit tumoral. La patiente est actuellement sous tamoxifène. Elle est en rémission complète après un recul de 20 mois.

## Discussion

Sur le plan embryologique, la glande mammaire dérive de l'ectoderme. L’épaississement de ce dernier forme la ligne lactée primitive ou crête mammaire étendue du creux axillaire au pli inguinal. Chez l’être humain, seuls deux bourgeons pectoraux persistent à la disparition de la crête mammaire à la 6ème semaine. A la naissance, la glande mammaire se limite à un court système de tubules, avec une aréole et un mamelon au sommet duquel s'ouvrent 15 à 20 canaux galactophores. Le développement se poursuit de façon lente pendant la période prépubertaire puis de façon plus rapide à la puberté en fonction des influences hormonales. La persistance de tissu mammaire ectopique est secondaire aux anomalies de développement embryologique [1, 2].

Le concept de sein ectopique peut être subdivisé en 2 types: un sein surnuméraire en cas de présence d'une glande persistante ou atrophique avec présence d'un mamelon et/ou d'une aréole, et un sein aberrant en cas de présence de tissu mammaire seul sans mamelon ni aréole [3, 4]. La fréquence du tissu mammaire ectopique dans la population générale est de 6% [1, 5, 6] et la survenue du cancer du sein sur tissu mammaire ectopique est rare en moyenne entre 0,2 à 0,6% de l'ensemble des cancers du sein [1, 3]. La localisation la plus fréquente du cancer sur tissu mammaire ectopique est la région axillaire (60-90%) selon les séries [1, 3], d'autres localisations peuvent être rencontrées: région para-sternale, sous claviculaire, sous mammaire et vulvaire [2, 3, 6, 7]. Au niveau de la région axillaire, le diagnostic différentiel se pose avec une adénopathie axillaire, il est alors étayé par la présence de tissu mammaire avec des canaux et des lobules adjacents au tissu tumoral et l'absence de tissu ganglionnaire lymphoïde, ceci permet d’éliminer une métastase ganglionnaire d'une tumeur mammaire occulte [1, 3]. L'extension ganglionnaire avoisine la moitié des cas, cette fréquence est due à la proximité entre la tumeur et les ganglions, et détient une valeur pronostique péjorative; le pronostic est aussi altéré par le retard diagnostique, vu que le mamelon ou l'aréole sont absents dans la majorité des cas, le tissu mammaire passe alors inaperçu en particulier s'il n'est pas en position axillaire [1, 2]. Cependant, à stade égal, le pronostic du cancer du sein sur tissu mammaire ectopique est identique au cancer du sein eutopique [3].

La prise en charge thérapeutique comporte une tumorectomie large avec un curage ganglionnaire axillaire homolatéral. Le traitement adjuvant est identique au cancer du sein. La technique du ganglion sentinelle est sujette à de nombreuses discussions vu la possibilité de drainage vers les ganglions axillaires controlatéraux ou même inguinaux. La rareté de cette pathologie ne permet pas d’établir des standards thérapeutiques [5].

## Conclusion

Le cancer du sein sur tissu mammaire ectopique est rare. Il pose un double problème diagnostique et thérapeutique. Il faut l’évoquer devant tout nodule sous cutané de diagnostic incertain situé à proximité de la ligne lactée en réalisant une échographie et une microbiopsie.
